# Age, Political Participation, and Political Context in Africa

**DOI:** 10.1093/geronb/gbae035

**Published:** 2024-03-18

**Authors:** Eugene Emeka Dim, Markus H Schafer

**Affiliations:** Department of Sociology, University of Toronto, Toronto, Ontario, Canada; Department of Sociology, Baylor University, Waco, Texas, USA

**Keywords:** Age and politics, Political activism, Political freedom, Political opportunity structure, Protesting

## Abstract

**Objectives:**

Political participation differs across the age range, but little is known about these patterns outside of developed countries. Political context is a particularly important consideration for all political behavior in Africa, where only a few countries are fully democratic. Drawing from political opportunity structures theory, we investigate how political freedom conditions the age-based pattern of electoral and nonelectoral political engagement, as well as protesting.

**Methods:**

This study merges the fifth, sixth, and seventh rounds of the Afrobarometer data sets, spanning 36 African countries, with country-level data on political freedom from Freedom House. Using multilevel regression models, we examine how political freedom shapes the relationship between age and 3 forms of political participation.

**Results:**

Africans aged from 18 to 60 years and living in nonfree countries are most engaged in electoral and nonelectoral political activities, though participation begins to drop markedly past age 60. For protest participation, young Africans living in partially and non-free countries are the most engaged in protests; yet limited political freedom again means a sharp age-based decline.

**Discussion:**

The impact of political context on the age–participation association is nuanced in ways not anticipated by mainstream research on the developed West. Repressive regimes, while spurring engagement at younger ages, appear to disproportionately deter older Africans from political engagement, especially its riskiest forms. We conclude by calling for more country-comparative gerontological research with careful attention to contextual heterogeneity, particularly in the understudied Global South.

Healthy democracies depend on widespread political participation among all members of society, including people of different age groups. Still, participation in a diverse range of political activities—from taking part in the electoral system to grass-roots mobilization—varies over the lifespan (Cutler, 1977; Melo & Stockemer, 2014; Nie et al., 1974; Quintelier, 2007; Stockemer & Rocher, 2017). At least among developed countries, the age pattern for most forms of political participation is consistent, rising from a trough in young adulthood, peaking in late middle age, and falling again at the end of life (Cutler, 1977; Melo & Stockemer, 2014; Nie et al., 1974). This phenomenon is perhaps best known in the realm of voting, where older adults’ turnout at the polls is seen as a leading explanation of “gerontocracy,” or the predominance of older elected leaders in most countries (Goerres, 2007; Magni-Berton & Panel, 2021). Similarly, older adults are disproportionately engaged in campaigning, attending party rallies, and working for political candidates (Melo & Stockemer, 2014). On the other hand, noninstitutionalized or “risky” forms of participation, including protesting and demonstrations, crest in young adulthood and then monotonically decline, becoming quite rare among people above retirement age (Melo & Stockemer, 2014; Nie et al., 1974).

Less understood, however, is how *country-level conditions* shape such age patterns of political participation (Serrat et al., 2020). Theoretical perspectives from political sociology underscore how political behavior is modified by the political context of a country—open political systems enabling but repressive regimes dampening most forms of political engagement (Dalton et al., 2009; Tilly & Tarrow, 2015). And though modern Western countries are marked by freedom of speech and of the press (Dalton et al., 2009), emerging democracies, particularly in the Global South, display much more variability in political freedom (Tilly & Tarrow, 2015). This set of countries offers an instructive counterpoint for understanding how responsive the rise and fall of political behavior across the age range is to political context. Do repressive conditions further accelerate the drop-off of protesting at an advanced age? Do they widen the gap between middle and older age adults when it comes to volunteering for political campaigns or organizing political efforts in the community? When repression makes even relatively mainstream forms of participation risky, it may have multifaceted implications for the participation age curve and cut short older adults’ role in political life.

The current study takes up these questions in the African context. Most research on the contextual impact of age on political behavior has been conducted using the United States or European samples, with other nations and world regions clearly underrepresented, if not absent altogether (Serrat et al., 2020). This paper, therefore, contributes to the existing literature by incorporating a neglected region of the Global South. Africa’s population is expected to double by 2050, likely encompassing one-third of the total world population by 2100 (Suzuki, 2019). And though it has a far younger population structure than the developed West, Africa’s projected growth rate of people aged 60+ is steeper than anywhere else in the world (He, 2022). This intersection of factors makes the representation of Africa in aging and political participation research an urgent objective for gerontologists. Thus, we use rounds five, six, and seven of the Afrobarometer survey, spanning 36 African countries, linked to Freedom House’s Freedom Index to investigate the impact of political context on the link between age and political participation.

## Political Participation Over the Lifespan

Political sociologists identify several strands of political participation. In general, political participation refers to the range of “activities by private citizens that are more or less directly aimed at influencing the selection of governmental personnel and/or the actions they take” (Verba et al., 1978, p. 46). Earlier literature on the concept distinguished between institutionalized and noninstitutionalized modes of political participation (Barnes & Kaase, 1979; Verba & Nie, 1972). Institutionalized participation includes activity centered within the electoral system and established institutions (Barnes & Kaase, 1979). These activities function according to the rules of conduct in established state law (Kaase, 1999; Marien et al., 2010). Examples include campaigning for electoral candidates, attending political party meetings, being a member of a political party, and working for political party candidates, among other activities. Noninstitutionalized participation consists of activities that challenge or defy institutions or the dominant culture (Barnes & Kaase, 1979). Activities reflecting noninstitutionalized participation include signing petitions, attending demonstrations, sit-ins, strike actions, and protesting, among others (it is important to note that noninstitutionalized forms of participation may or may not be legal, depending on the levels of freedom and civil liberties in a particular country). Some of these more confrontational forms of engagement can be classified as “risky,” depending on the particular laws of a given country and its tolerance of oppositional action (Caren et al., 2011).

Scholars draw upon multiple theoretical explanations to account for why these varied dimensions of political participation would vary by age. According to the *life-cycle model*, advancing age leads to an accumulation of roles and resources that boost older people’s stakes in the political process (Nie et al., 1974). The preservation of old-age entitlement programs, meanwhile, makes older voters an important constituency to be courted by political parties (Walker, 2006). Conversely, younger people tend to be more geographically mobile and less rooted in a community, less wealthy, and lacking the children (and grandchildren) whose future motivates political engagement (Jankowski & Strate, 1995). Another theoretical explanation centers on attitudes, particularly institutional trust, and cynicism (Quintelier, 2007). Younger people, for instance, are relatively unlikely to see the established political class—comprised disproportionately of older citizens—as trustworthy, responsive, and in touch with their needs (Henn et al., 2002; O’Toole et al., 2003). Parenthetically, we note that most empirical studies used to support these age-based theories rely on cross-sectional age comparisons, rather than within-person, longitudinal change (e.g., Goerres, 2007, 2009; Melo & Stockemer, 2014; Panagopoulos & Abrajano, 2013; Quintelier, 2007). The same will be true for our analysis below.

Together, the narrative of life-cycle difference and disillusionment could simply suggest younger adults are less politically engaged than older citizens. Still, other observers note that such explanations often fail to encompass the full spectrum of noninstitutionalized participation (Melo & Stockemer, 2014). Indeed, careful analyses conceiving of “the political” with adequate breadth—including activity targeting post-materialist ends, such as self-expression, equal rights, and environmental concerns (Inglehart, 1997)—tend to find less of a participation gap between the young and the old (Marien et al., 2010; Melo & Stockemer, 2014; Quintelier, 2007). Furthermore, much of this cause-oriented participation is facilitated through social networks, increasingly mobilized through online channels (Vissers & Stolle, 2014); this too tends to incorporate younger citizens into the political process. Attending to political participation in its full diversity, therefore, leads scholars to de-emphasize a single linear pattern of age-based decline.

## The Political Context of Aging and Participation

An additional theoretical framework with potential explanatory power across the lifespan highlights the importance of resources and barriers (Verba et al., 1995). Indeed, this resource model approach is prominent in gerontology, helping identify variation in education and political interest, free time, and health as predictors of how often people contact elected representatives or work in voter turnout efforts (Campbell & Binstcok, 2011; Serrat et al., 2017; Walker, 2006).

An oversight of research on resources and barriers, however, is the nearly exclusive focus on individual-level characteristics, alongside an implicit assumption that aging adults are participating in a free and open political context. *Political opportunity structures theory* in sociology challenges this orientation, emphasizing that participation is shaped by the broader set of political constraints and opportunities unique to the environments in which people are embedded (McAdam, 1999; Meyer, 2004). Perhaps the most critical feature in this regard is the *openness of the political system*, the extent to which individuals can access the polity (Tilly & Tarrow, 2015). Authoritarian or politically closed political systems tend to suppress engagement, especially noninstitutionalized forms of participation (Marien et al., 2010). Still, research on age and political participation has seldom considered these types of contextual contingencies.

One of the rare studies to consider how political context shapes age-based political participation found that among 27 European countries, the level of participation in institutionalized politics for people in their 20s, 30s, and 40s was depressed in countries with a legacy of authoritarian regimes (Hooghe & Quintelier, 2014). Even so, attention to the adults across the age span, *particularly those in old age*, remains understudied—particularly in emerging democracies in the Global South.

## Political Participation Across the Lifespan in Africa

The role of political context is especially important in Africa, home to many emerging democracies with unique sociodemographic and political challenges. Numerous African countries are within their early stages of political development, featuring low levels of socioeconomic prosperity (Arvis et al., 2018; UNDP, 2019). Different measures of political freedom describe most African countries as politically volatile and unstable—even sometimes as failed states (Economic Intelligence Unit, 2021; Repucci & Slipowitz, 2022; Vasquez et al., 2021). Only a small handful of African countries are fully democratic. There have been about 20 coups per decade from 1960 to 2000 across the continent, many of which have led to tyrannical regimes, disrupted democracy, political instability, and further socioeconomic and political underdevelopment. The life-course timing of these events could produce long-lasting changes in the willingness of older adults to participate in politics, especially if there are politically formative years when unrest is most indelible (Marien et al., 2010). Such potential cohort effects are challenging to disentangle from age effects, particularly with the finite number of countries and historical trajectories in our data. It is rare, for instance, for countries exhibiting high instability in the 1960s to have avoided repression in all the decades that followed, remaining open and democratic countries by the study period (only Ghana and Tunisia may fit this profile). Rarer still are countries shifting from free to unfree or partially free for a bounded time and then back to free (like Lesotho), or who have been free for the latter half of the twentieth century but become unfree or partially free in our observational window (like Mali).

When it comes to age, scholars note the low voter turnout among young people (ages 18–30) across Africa (Resnick & Casale, 2011, 2014). Compared to people aged 30+, young Africans also express lower levels of political partisanship and overall interest (Chikwana & Masunnungure, 2007)—and, perhaps surprisingly, report protesting at comparable rates (Resnick & Casale, 2011). Though this latter finding may call into question the typical assumption that younger people substitute noninstitutionalized engagement for more institutionalized forms, the dichotomization of age (30 and under vs over) in these early analyses obscures the nature of political participation age curves, especially at the more advanced years.

## Research Questions

Merging existing knowledge about age and political participation in Arica and beyond with insights from political opportunity theory, this study raises the following questions:

1. What is the overall relationship between age and political participation patterns among Africans, and do these patterns differ by form of political engagement (i.e., institutionalized vs noninstitutionalized)?
2. Do low levels of political freedom modify age differences in political engagement, especially at the higher end of the age range? We hypothesize that in low-freedom contexts, there will be a less pronounced rise in institutionalized participation across mid to late adulthood than in high-freedom countries, owing to governments’ lack of political responsiveness to the needs and interests that typically prompt such engagement. We also hypothesize that in such contexts, noninstitutionalized participation will dip at earlier ages and in a more pronounced fashion, owing to the riskiness of grass-roots mobilization under repressive regimes.

## Method

Data for this study come from multiple rounds of the Afrobarometer, collected from 2011 to 2018. The Afrobarometer is a multicountry survey project undertaken on the socioeconomic and political behaviors, attitudes, and perceptions among adult Africans. The fifth (2011–2013), sixth (2014–2015), and seventh (2016–2018) rounds of the Afrobarometer surveys were combined for the analyses. The data set covers over 150,000 respondents from 36 countries (see Supplementary Table 1). The choice of the Afrobarometer surveys is appropriate for this study as they provide the most comprehensive account of the political behavior and attitudes of adult Africans, with a relatively high response rate and sample size.

The samples selected from each country of the surveys range from 1,200 to 2,400 participants (with response rates ranging from 70% to 90%), respondents representing a probabilistic sample of each country’s voting population. Clustered, stratified, multistage, and area probability sampling were combined to create the sample design. The sample size for each country was stratified based on the main subnational unit of the state and by the rural–urban area of residence in each country.

### Dependent Variables

Outcome variables for this study capture political participation. We employed the full range of political activities gathered in the Afrobarometer rounds used. Measures refer to activities undertaken in the past year and include the following: attending community meetings, raising an issue during community meetings, attending protests or demonstrations, being affiliated with a political party, attending political rallies, and working for political candidates. We coded each activity as 0 (did not participate) and 1 (participated). After an exploratory factor analysis, we created an additive index for electoral participation (EP) and nonelectoral participation (NEP) (the analysis applied a polychoric correlation matric using a nonorthogonal promax rotation [Holgado-Tello et al., 2010]). Corresponding with that analysis and aligning with the literature, we added attending political rallies, working for political candidates, and being affiliated with a political party to constitute electoral participation. For nonelectoral participation, we added the items for attending community meetings and raising an issue in the community. We opted for an additive scale to ensure the easiest interpretability. To minimize missing data, we counted as valid any outcome score where the respondent responded to at least one of the relevant questions. Respondents who missed two or three items were counted as missing (*N* = 12,821). Those missing were disproportionately women, unemployed, less educated, less interested in politics, young, not members of voluntary associations, and rural. As a sensitivity analysis, we estimated all models for outcome variables generated by the polychoric factor scores. Results were essentially identical to those presented in our tables and figures. Protesting was separated from the other measures to be used as a separate, higher-risk dimension of political engagement (McAdam, 1986); it also did not load well on either factor encompassing the five other forms of participation. The factor loadings are shown in Supplementary Table 3.

### Independent and Moderating Variables

Age is the focal independent variable, measured as a count of years since birth year. The variable was also squared to capture potential nonlinear associations between age and political participation.

We combined country-level variables with the individual- level Afrobarometer data. The focal contextual variable for this study is political freedom, measured with the freedom index developed by Freedom House. The freedom index classifies countries into “not free,” “partly free,” and “free,” and we adopt that categorization, following previous research (Puddington, 2013). The freedom index is derived from several indicators, such as the level of transparency in electing the head of state and legislators; the level of freedom in partisan politics; the level of functioning and transparency of the government; the level of freedom of political expression; the level of freedom of assembly; the adherence of the rule of law; and the level of personal autonomy and individual rights. A table of countries by their freedom index is included in Supplementary Material. We use freedom index scores from 2010 for round five, 2013 for round six, and 2016 for round seven.

### Control Variables

Individual-level control variables for this study include gender (0 = *Male*, 1 = *Female*), educational attainment (0 = *None*/*Informal*, 1 = *Primary*, 2 = *Secondary*, 3 = *University*), employment status (0 = *Unemployed*, 1 = *Employed*), membership in voluntary association (0 = *Not a member*, 1 = *Inactive*, 2 = *Active*), political interest (0 = *None*, 1 = *Not very interested*, 2 = *Somewhat interested,* 3 =* Very interested*), and place of residence (0 = *Rural*, 1 = *Urban*). Country-level control variables include logged gross national income per capita (current U.S. dollars) as reported by the World Bank and system of government (0 = *Presidential system*, 1 = *Semi-Presidential*, 2 = *Parliamentary*). The waves of the study were also added as a control variable to account for period effects.

#### Method of analysis

We used hierarchical linear models (HLM) to analyze the impact of political context on the association between age and political participation. HLM is suitable for this study as it can be used to examine cross-national variation in an outcome variable while capturing within-country differences among respondents in their countries (Raudenbush & Bryk, 2002). Three HLMs were estimated, that is, one for each form of political participation. Linear HLM was used to analyze EP and NEP. As counts of political activities, Poisson or negative binomial estimators are plausible alternatives to the linear HLM. However, we found that the conditional mean was higher than the conditional variance for both outcomes, thus making Linear HLM appropriate for the analysis. Logit HLM was used to analyze protesting, as it is a dichotomous variable. Additionally, we include a random coefficient for age, enabling us to assess the possible varying effect of age across the countries.

Prior to fitting HLMs, we analyzed intraclass correlation (ICC) coefficients with a one-way analysis of variance (ANOVA) across the dimensions of political participation. ICC coefficients reveal that the proportion of variance in political participation accounted for by nation-level differences varied across outcomes. Specifically, between 6% and 10% of the total differences in political participation occur because of country differences.

Our results section presents six HLMs. Models 1, 3, and 5 show the influence of age on EP, NEP, and protesting, respectively. Models 2, 4, and 6 incorporate cross-level interaction effects of age and freedom index on the three outcomes. This sequence of models shows whether political structures modify the cross-national difference in political participation age patterns. We derived predicted mean scores (EP, NEP) and probabilities (protesting) from the interaction models for ease of interpretation and to aid with statistical inference. Given the inability to compare interaction terms in logit models (Mood, 2010), average marginal effects (AME) were conducted for the interaction effects of age and political context on protesting. We produced AME estimates for the first and second differences in testing the equality of the marginal effects for age and freedom index (Mize, 2019).

## Findings

Table 1 shows descriptive results for study variables. The table shows that Africans, on average, engaged in 1.07 (of the possible two measured) electoral activities while participating in 1.05 out of three nonelectoral activities. That said, most Africans were engaged in at least one form of electoral (65%) and nonelectoral (64%) activity. Only about 10% of the overall sample participated in protests in the past year. The mean age of the respondents was 37.2. In terms of political context, the average score of the freedom index from 2010 to 2015 for African countries is 1.06, as most of the African countries (58.5%) were fairly free. About 17.5% of the countries are not politically free, and 24% were free countries.

**Table 1.** Focal, Control and Outcome Variables of the Afrobarometer Data

Individual-level variable	Mean	*SD*	Proportion	Minimum	Maximum
Age	37.21	14.68		18	106
Gender (women)	0.50	0.50		0	1
Educational level	1.03	0.96		0	3
Urban residence	0.42	0.49		0	1
Voluntary group membership	0.59	0.84		0	2
Employment status	0.59	0.84		0	2
Political interest	1.39	1.07		0	3
Country variables					
Freedom status					
Not free			17.5%	0	1
Partly free			58.5%	0	1
Free			24.0%	0	1
Gross national income per capital (logged)	8.07	0.86		6.57	10.04
System of government					
Presidential			70.0%	0	1
Semi-Presidential			16.4%	0	1
Parliamentary			11.2%	0	1
Monarchy			2.4%	0	1
Outcome variables					
Electoral Participation (EP)	1.07	0.98		0	3
Nonelectoral participation (NEP)	1.05	0.89		0	2
Protesting	0.10	0.30		0	1

### Age and Political Participation

**Figure 1.**
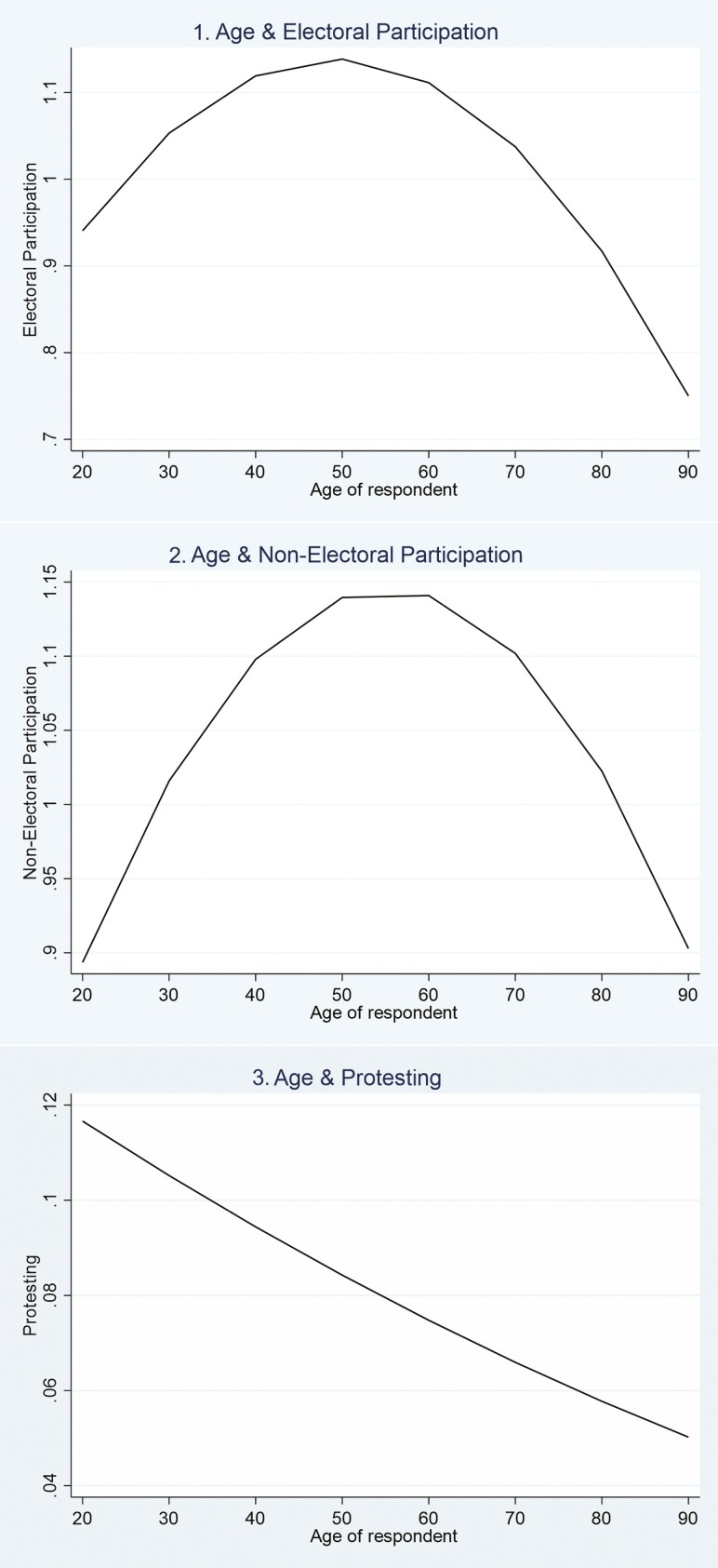
Associations between age and the three dimensions of political participation. The predicted scores for Figure 1 were derived from Models 1, 3, and 5 in Table 2.

Table 2 shows the associations between age and the three dimensions of political participation used in this study. As shown in models without cross-level effects, age had a significant nonlinear association with EP and NEP (models 1 and 3). Figure 1 (models 1 and 2), using margin scores generated from those models, clarifies the concave shape and shows that both forms of behavior peak around age 50. For NEP, each additional year is associated with more political activity through the range of young adult ages, whereas electoral activity starts from a relatively high level among those in their early 20s, increases gradually for those in their 30s and 40s, and then declines. Protesting, however, takes a different form, being highest among young adults and dropping consistently across the age range.

**Table 2.** Multilevel Analyses of Age, Political Context, and Political Participation

Variable	Electoral participation (linear regression) (*n* = 146,010)		Non-electoral participation (linear regression) (*n* = 145,705)		Protest (logistic regression) (*n *= 143,604)	
	Model 1	Model 2	Model 3	Model 4	Model 5	Model 6
	Coef. (*SE*)	Coef. (*SE*)	Coef. (*SE*)	Coef. (*SE*)	Coef. (*SE*)	Coef. (*SE*)
Freedom Index						
Not free (ref.)						
Fairly free	−0.11*** (0.01)	−0.18*** (−0.03)	−0.04** (0.01)	−0.10** (0.03)	0.17** (0.07)	−0.15 (0.14)
Free	−0.18*** (0.01)	−0.26*** (−0.04)	−0.14*** (0.01)	−0.20*** (0.04)	−0.20** (0.07)	−0.49** (0.15)
Age	0.02*** (0.001)	0.02*** (0.001)	0.02*** (0.001)	0.02*** (0.001)	−0.006 (0.004)	0.01** (0.004)
Age-squared	−0.0002*** (0.00002)	−0.0002*** (−0.0002***)	−0.0002*** (0.000001)	−0.0002*** (0.00001)	−0.00007^a^ (0.00004)	−0.00007^a^ (0.00004)
Age × freedom status						
Age × not free (ref.)						
Age × partly free		0.002* (0.0008)		0.002* (0.0007)		0.009* (0.003)
Age × free		0.002* (0.001)		0.002^a^ (0.0008)		0.008* (0.003)
Intercept	3.44*** (0.29)	3.52*** (0.29)	2.28*** (0.21)	2.33*** (0.21)	−3.46*** (0.75)	−3.26*** (0.78)
Random effects	0.28 (0.03)	0.29 (0.04)	0.20 (0.03)	0.20 (0.03)	0.72 (0.1)	0.70 (0.10)
Random effects (age)	0.004 (0.0005)	0.004 (0.001)	0.003 (0.0004)	0.003 (0.0004)	0.01 (0.001)	0.01 (0.001)

*Notes: SE* = standard error; Ref. = reference group.

Model applied robust standard errors. Models control for rounds of the study, gender, education, urban residence, membership in a voluntary organization, employment status, political interest, GNI (logged), and system of government.

^a^ *p* < .06.

*p < .05, **p < .01, ***p < .001.

**Figure 2.**
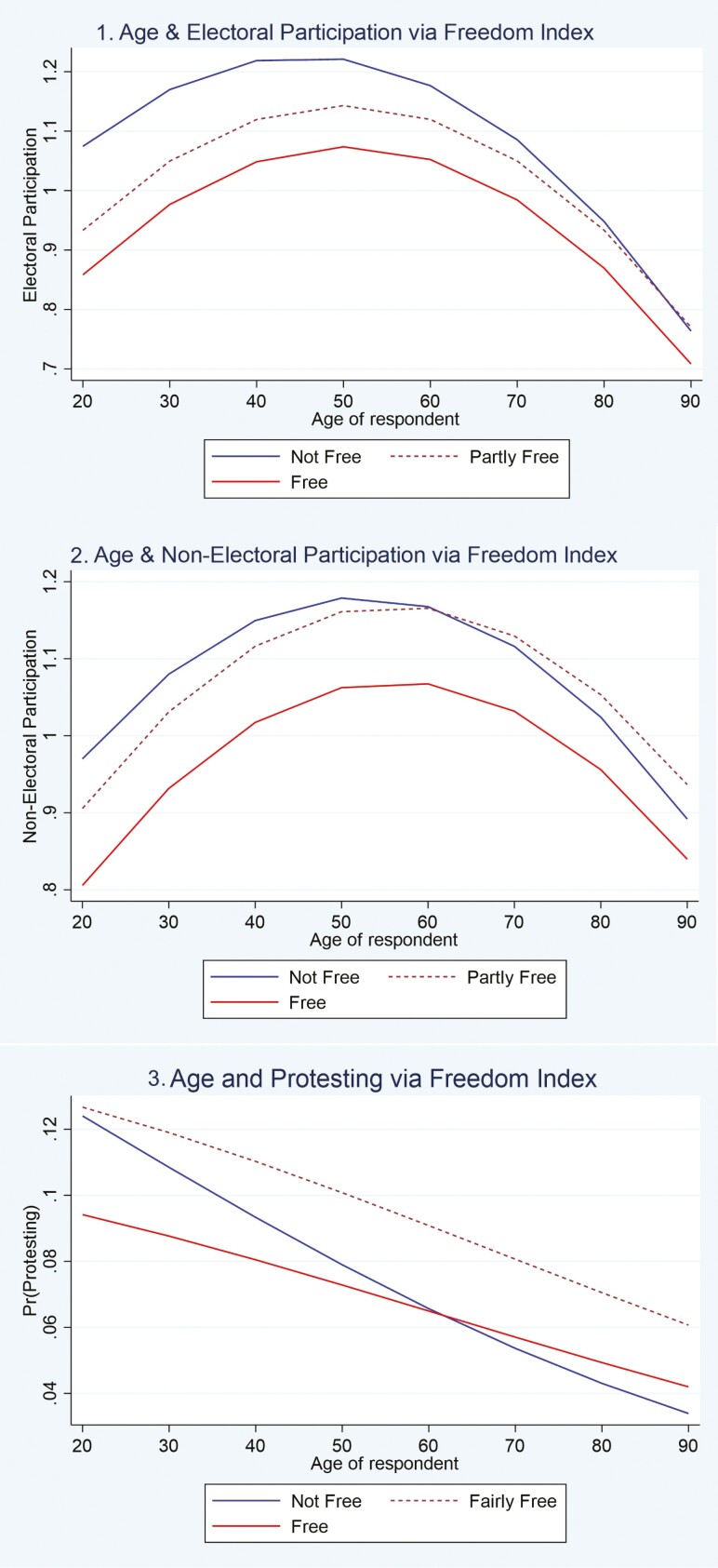
Margin plots of age, political context, and participation. The predicted scores for Figures 1 and 2 were derived from Models 2, 4, and 6 in Table 2.

Models 2, 4, and 6 introduce cross-level interaction effects of age and political freedom (the full table for the models is shown in Supplementary Table 2). Specification of these models was determined by considering Akaike and Bayesian Information Criterion; from preliminary analyses, we found that models interacting age by political context fit better than models including age^2^ × political context. These analyses helped indicate that the slopes of the age parabola differed by political context, but not the curvature of age with the three outcomes. From model 2, the concave-shaped link between age and EP differs by political freedom. Figure 2 clarifies that Africans aged from 18 to 50 years living in nonfree countries are most engaged in electoral activities (with peak activity around 40 and 50 years); however, drop-offs in participation across the age distribution are most pronounced for those living in nonfree contexts. For instance, a 40-year-old in a nonfree country, like Gabon, would be predicted to engage in 0.59 of three electoral activities, compared to 0.45 for a 70-year-old (i.e., [0.45–0.59]/0.59 = 24% decrease). The comparable drop by age in free countries, such as Mauritius, is from 0.40 for 40-year-olds to 0.34 for 70-year-olds (i.e., a 15% decrease).

For NEP (Figure 2), most Africans in nonfree countries again show the highest rates of activity (with peak activity at 50 years). This remains consistent until the age of 60, when participation starts to drop to levels similar to those living in fairly free countries. Africans living in free counties are the least engaged in nonelectoral activities across the full age span.

For protesting behavior, Africans living in partially free countries are the most engaged in protests, this distinction holds up across all segments of the adult age range. The negative association between age and protesting is apparent in all three types of political contexts, but it is most pronounced in the nonfree countries. The tendency for protests is similar for young people living in nonfree and fairly free countries, and their counterparts in free countries protest less. However, among individuals above 70 years, protest is least common in nonfree countries and highest in fairly free countries. The gap, as shown in Figure 2, is as large as 16%. Table 3 shows that these context-specific age patterns are statistically significant. For any given age in the distribution, the percentage point drop for a five-year increase is 0.09 in nonfree countries versus 0.05 in free countries, a second difference of 0.04 (*p* < .05). Moving across specific 15-year age spans, the likelihood of protesting falls between ages 52 and 67 in nonfree countries by 0.33 probability points, but only by 0.2 in fairly free and by 0.21 in free countries. Similarly, the likelihood of protesting falls between ages 67 and 82 in nonfree countries by 0.36, but only by 0.23 in fairly free and by 0.24 in free countries. The second-difference scores associated with the age drop-offs in nonfree versus free countries are all statistically significant (*p* < .05). Second-difference scores confirm that the age drop-off between free and fairly free contexts, however, are not statistically different from one another.

**Table 3.** Results for How Protest is Associated with Age (age-squared) and Freedom Status: Tests of Average Marginal Effects (AMEs) and Second Differences

Effect of every 5 y	AME_non-free_	AME_fairly free_	AME_free_	Second Diff.^1^	Second Diff.^2^	Second Diff.^3^
Start_i_ + 5	−0.093***	−0.050***	−0.054***	0.043***	−0.004	0.039*
Effects of age groups						
22→37	−0.258***	−0.127***	−0.137**	0.131*	−0.009	0.121*
37→52	−0.292***	−0.162***	−0.171***	0.131*	−0.009	0.121*
52→67	−0.327***	−0.196***	−0.206***	0.131*	−0.009	0.121*
67→82	−0.361***	−0.230***	−0.240***	0.131*	−0.009	0.121*

*Notes*: Second Diff.^1^ = Not free to Fairly free; Second Diff.^2^ = Fairly free to Free; Second Diff.^3^ = Not free to Free.

The choice of the age group gaps was made based on the standard deviation of age (i.e., 15).

*p < .05, **p < .01, ***p < .001.

## Discussion

This study examines how lifespan patterns of political participation are refracted through political context. Our analysis featured 36 African countries—an approach that accentuates an understudied region and that leverages key variability in political opportunity structure. Our first question was simply whether the age-based patterns documented so consistently in advanced Western democracies would replicate in Africa. The second question, informed by theory in political sociology, sought to understand if levels of political freedom modify age differences in political participation, particularly whether repression hastens drop-offs at older ages.

For the first question, we found that middle-aged Africans, especially those between the ages of 40 and 60 years, were the most involved in politics, both electoral and nonelectoral. This is consistent with life-cycle explanations of political behavior—that it takes time for adults to accumulate the resources, life experiences, community connectedness, and other incentives to be most active in the political process (Nie et al., 1974). We note that the lack of an available indicator of voting may have dampened the overall prevalence of electoral participation for older adults in this study and led to an overestimate of late-life decline relative to young adulthood. Nevertheless, older people, especially those in their 60s, 70s, and 80s, were considerably more engaged in nonelectoral participation than younger people. This more strongly affirms the claims of the life-cycle model. Young people, by contrast, were by far the most likely to have recently protested, and the rate of decline in this form of activity (inferred by age differences observed cross-sectionally) was pronounced. This is consistent with the claim that young people are most geographically mobile, the least wealthy, and lack key social ties that would inhibit their engagement in risky political engagement (Jankowski & Strate, 1995). Young Africans also appear most distrustful of the political class, which could motivate contentious action (see Henn & Foard, 2011). Further analysis of the Afrobarometer data revealed that young people are more likely to distrust the President, the police, and the legislature in their respective countries (results available upon request).

Our second—and primary—research question addressed contextual contingency. We found that the impact of political context on age–participation association is nuanced, departing somewhat from existing versions of political opportunity structure theory. First, for institutionalized and noninstitutionalized political activity, engagement is more pronounced—especially for young and middle-aged Africans—in nonfree countries. This finding differs from previous studies conducted in different settings (Henn & Foard, 2011; Hooghe & Quintelier, 2014). These studies suggest that young people’s participation in institutionalized politics is depressed in closed political systems or authoritarian regimes. Possibly, the high levels of electoral participation for young and middle-aged Africans in nonfree states reflect a desire to seek better socioeconomic conditions through the democratic process. On the flip side, the persistent pattern of relatively low participation in free countries across the age range may imply that people living in a fully democratic setting feel a lower impetus to engage, perhaps due to a sense of having already achieved what is not allowed by neighboring authoritarian and military governments. The economic prosperity fostered by democratization could motivate individuals to pursue other forms of political expression not captured in the Afrobarometer data set (Acemoglu et al., 2019).

That said, rates of both institutionalized and noninstitutionalized participation decline most precipitously by age in the nonfree contexts. This accords with our expectations and suggests a pattern of late-life political disengagement in repressive countries. This could result from disillusionment and burnout from socioeconomic conditions not changing for the better. Africans in nonfree countries may become disoriented with their political system due to an inability to gain many of the stakes in a society that would otherwise motivate continued political engagement. There is also the danger and risk of political repression and persecution. Future research is needed to test the relative importance of these two mechanisms.

Our final outcome, protesting in the past year, also reveals more complexity than foreshadowed by literature from the developed West. Indeed, protesting is more prevalent in *fairly free* countries across the full age range. Partially free countries are political systems between a full democracy or an authoritarian regime, often representing a shift from one to the other. Many of these countries, such as Nigeria and Burkina Faso, have transitioned from colonization or military authoritarian regimes to hybrid democracies, retaining some marks of autocracy and remaining susceptible to political instability, corruption, human rights violations, and mass violence (Bogaards, 2009; Diamond, 2002; Quigley, 1983). In these countries, ambitious soldiers tend to either legitimize their rule by running for civilian leadership in “contested, multiparty elections” (that tend to be violent, fraudulent, and coerced), or carve out large, autonomous domains of political and economic dominance under the veil of civilian rule (Diamond, 2002, p. 27). Africans in such countries may be motivated to fight for the civil and political freedoms they lack. All the same, these pursuits are undoubtedly risky in both partly free and (especially) nonfree countries. This point is underscored by the notably distinct drop-off by age in the latter set of countries.

This study has several limitations. First, the range of political participation indicators is limited by the political activities measured in the last three rounds of the Afrobarometer data sets. Most significantly, voting was not included in this study due to the lack of consistency for that measure among the countries in our sample. That is, not all countries conducted elections during the years 2010, 2013, and 2015. We recognize that older people tend to be more active in voting than younger people, and this would have implications for interpreting electoral participation by age. We are also cognizant of other unmeasured activities that would provide a more exhaustive depiction of political participation; these include engaging in strike actions, donating to political candidates, contacting public office holders and legislators, and signing online and in-person petitions, among others. Nevertheless, we believe that the indicators used in this study capture the essence of institutionalized and noninstitutionalized political activity. Future surveys may include a more robust range of political participation indicators. Another set of measures we would hope to see in future rounds of the Afrobarometer surveys would be those related to health. For instance, physical functioning could plausibly mediate age effects, a process beyond the scope of the current analysis. Further research likewise invites the opportunity to explore other potential moderators of the age and political participation association, including context-based ageism and individual motivation.

Finally, we are keenly aware that our interpretation of age and lifespan patterns may be picking up on cohort differences. Age and cohort are inextricably linked in this research design, and longer-running repeated cross-sections would be needed to disentangle these two issues more convincingly. With only 36 African countries in the Afrobarometer survey, there are not enough degrees of freedom to statistically estimate how different levels of current political freedom combine with unique historical legacies, that is, enabling enough variation in life-course exposure to tease out distinctive cohort patterns. Future longitudinal studies could focus on countries that have transited from authoritarianism or partial democracies to open systems and full democracies. Such approaches could help clarify how people of different ages engage with the political system as the country’s democratization process evolves. Overall, however, we emphasize that the observational form of these or similar data limits any strong causal conclusions.

These limitations considered, the present study breaks important new ground by bringing political opportunity structure theory to the context of Africa, a diverse and rapidly aging region still overlooked by most gerontologists (Serrat et al., 2020). This study reveals important insights about political agency across the lifespan. Despite relatively strong participation among young adults, Africans living in nonfree polities show the steepest drop-off in electoral and nonelectoral political activities past age 60. Repressive regimes appear to especially deter older adults from the riskiest forms of participation. In sum, this suggests that many older adults on the African continent are being disproportionately excluded from political life, even as this segment of the population stands to surge in the near future (He, 2022). Political context can shape a host of age-related political incentives, attitudes, and motivations, and more research is needed—in Africa and in other areas of the Global South—to further understand how these dynamics of political participation over the lifespan differ or align with patterns well-known in developed countries.

## Supplementary Material

gbae035_suppl_Supplementary_Tables_1-3
