# Supplementary material for: Age, Political Participation, and Political Context in Africa
Source: J Gerontol B Psychol Sci Soc Sci. 2024 Mar 18;79(6):gbae035. doi: 10.1093/geronb/gbae035 (PMC11077311; doi:10.1093/geronb/gbae035)
Supplement: gbae035_suppl_Supplementary_Tables_1-3 [file gbae035_suppl_supplementary_tables_1-3.docx]

**Supplementary Material**

**Supplementary Table 1: Political participation and freedom index score by country**

| **Countries** | **Electoral participation** | **Non-electoral participation** | **Protesting** | **Freedom Index score** |
| --- | --- | --- | --- | --- |
| Algeria | 0.28 | 0.97 | 0.07 | 0 |
| Benin | 0.89 | 1.43 | 0.11 | 2 |
| Botswana | 0.52 | 0.61 | 0.11 | 2 |
| Burkina Faso | 0.61 | 1.63 | 0.14 | 1 |
| Burundi | 0.57 | 2.02 | 0.04 | 1 |
| Cameroun | 0.47 | 1.56 | 0.11 | 0 |
| Cape Verde | 0.65 | 1.18 | 0.15 | 1.33 |
| Cote d’voire | 0.41 | 1.63 | 0.09 | 0.67 |
| Egypt | 0.18 | 0.65 | 0.13 | 0 |
| Gabon | 0.68 | 1.13 | 0.17 | 0 |
| Ghana | 0.45 | 1.40 | 0.06 | 2 |
| Guinea | 0.73 | 1.89 | 0.08 | 1 |
| Kenya | 0.60 | 1.78 | 0.09 | 1 |
| Lesotho | 0.58 | 1.90 | 0.10 | 1.33 |
| Liberia | 0.71 | 1.94 | 0.13 | 1 |
| Madagascar | 0.19 | 1.38 | 0.03 | 1 |
| Malawi | 0.75 | 2.33 | 0.05 | 1 |
| Mali | 0.29 | 1.61 | 0.09 | 1.33 |
| Mauritius | 0.29 | 0.76 | 0.05 | 2 |
| Morocco | 0.25 | 0.85 | 0.13 | 1 |
| Mozambique | 0.54 | 1.86 | 0.11 | 1 |
| Namibia | 0.40 | 1.56 | 0.11 | 1.67 |
| Niger | 0.64 | 1.76 | 0.06 | 1 |
| Nigeria | 0.41 | 1.43 | 0.14 | 1 |
| Sao Tome | 0.91 | 1.18 | 0.12 | 2 |
| Senegal | 0.72 | 1.65 | 0.14 | 1.67 |
| Sierra Leone | 0.71 | 0.74 | 0.06 | 1 |
| South Africa | 0.74 | 1.58 | 0.18 | 1.72 |
| Sudan | 0.39 | 1.33 | 0.14 | 0 |
| Swaziland | 0.30 | 1.18 | 0.07 | 0 |
| Tanzania | 0.86 | 2.03 | 0.08 | 1 |
| Togo | 0.54 | 1.39 | 0.12 | 1 |
| Tunisia | 0.16 | 0.64 | 0.12 | 1.33 |
| Uganda | 0.81 | 2.01 | 0.08 | 0.8 |
| Zambia | 0.56 | 1.46 | 0.05 | 1 |
| Zimbabwe | 0.63 | 1.74 | 0.04 | 0.2 |
| **Total** | **0.55** | **1.57** | **0.10** | **1.06** |

**Supplementary Table 2: Multilevel analyses of Age, Political context, and Political participation (all covariates)**

| **Variables** | **Electoral Participation** | | | | **Non-Electoral Participation** | | | | |
| --- | --- | --- | --- | --- | --- | --- | --- | --- | --- |
|  | **Model 1** | | **Model 2** | | **Model 3** | | **Model 4** | | |
|  | **Coef.** | **SE** | **Coef.** | **SE** | **Coef.** | **SE** | **Coef.** | **SE** |  |
| **Freedom Index** |  |  |  |  |  |  |  |  |  |
| Not free (Ref) |  |  |  |  |  |  |  |  |  |
| Fairly free | -0.11*** | 0.01 | -0.18*** | -0.03 | -0.04** | 0.01 | -0.10** | 0.03 |  |
| Free | -0.18*** | 0.01 | -0.26*** | -0.04 | -0.14*** | 0.01 | -0.20*** | 0.04 |  |
| **Age** | 0.02*** | 0.001 | 0.02*** | 0.001 | 0.02*** | 0.001 | 0.02*** | 0.001 |  |
| **Age-squared** | -0.0002*** | 0.00002 | -0.0002*** | -0.0002 | -0.0002*** | 0.000001 | -0.0002*** | 0.00001 |  |
| **Age X Freedom status** |  |  |  |  |  |  |  |  |  |
| Age X Not Free (Ref) |  |  |  |  |  |  |  |  |  |
| Age X Partly Free |  |  | 0.002* | 0.0008 |  |  | 0.002* | 0.0007 |  |
| Age X Free |  |  | 0.002* | 0.001 |  |  | 0.002^†^ | 0.0008 |  |
| **Study round** |  |  |  |  |  |  |  |  |  |
| Round 5 (Ref) |  |  |  |  |  |  |  |  |  |
| Round 6 | 0.06*** | 0.01 | 0.06*** | 0.01 | -0.10*** | 0.01 | -0.10*** | 0.01 |  |
| Round 7 | 0.23*** | 0.01 | 0.23*** | 0.01 | 0.10*** | 0.01 | 0.10*** | 0.01 |  |
| **Gender** |  |  |  |  |  |  |  |  |  |
| Male (Ref) |  |  |  |  |  |  |  |  |  |
| Female | -0.18*** | 0.004 | -0.18*** | 0.004 | -0.16*** | 0.004 | -0.16*** | 0.004 |  |
| **Education** |  |  |  |  |  |  |  |  |  |
| None (Ref) |  |  |  |  |  |  |  |  |  |
| Primary | 0.07*** | 0.01 | 0.07*** | 0.01 | 0.05*** | 0.01 | 0.05*** | 0.01 |  |
| Secondary | 0.03*** | 0.01 | 0.03*** | 0.01 | 0.04*** | 0.01 | 0.04*** | 0.01 |  |
| University | -0.02* | 0.01 | -0.03** | 0.01 | 0.05*** | 0.01 | 0.05*** | 0.01 |  |
| **Place of residence** |  |  |  |  |  |  |  |  |  |
| Rural residence (Ref) |  |  |  |  |  |  |  |  |  |
| Urban residence | -0.14*** | 0.01 | -0.14*** | 0.01 | -0.19*** | 0.004 | -0.19*** | 0.004 |  |
| **Voluntary association** |  |  |  |  |  |  |  |  |  |
| Not a member (Ref) |  |  |  |  |  |  |  |  |  |
| Inactive | 0.21*** | 0.01 | 0.21*** | 0.01 | 0.48*** | 0.01 | 0.48*** | 0.01 |  |
| Active | 0.35*** | 0.01 | 0.36*** | 0.01 | 0.69*** | 0.01 | 0.69*** | 0.01 |  |
| **Employed status** |  |  |  |  |  |  |  |  |  |
| Unemployed (Ref) |  |  |  |  |  |  |  |  |  |
| Employed | 0.04*** | 0.01 | 0.04*** | 0.01 | 0.01* | 0.004 | 0.01* | 0.005 |  |
| **Political interest** |  |  |  |  |  |  |  |  |  |
| Not interested (Ref) |  |  |  |  |  |  |  |  |  |
| Not very interested | 0.21*** | 0.01 | 0.21*** | 0.01 | 0.11*** | 0.01 | 0.11*** | 0.01 |  |
| Somewhat interested | 0.41*** | 0.01 | 0.41*** | 0.01 | 0.20*** | 0.01 | 0.20*** | 0.01 |  |
| Very interested | 0.60*** | 0.01 | 0.60*** | 0.01 | 0.29*** | 0.01 | 0.29*** | 0.01 |  |
| Log GNI | -0.40*** |  | -0.40*** | 0.04 | -0.24*** | 0.03 | -0.24*** | 0.03 |  |
| **System of government** |  |  |  |  |  |  |  |  |  |
| Presidential (Ref) |  |  |  |  |  |  |  |  |  |
| Semi-Parliamentary | 0.16 | 0.12 | 0.15 | 0.13 | 0.03 | 0.08 | 0.03 | 0.01 |  |
| Parliamentary | 0.34* | 0.14 | 0.34* | 0.15 | 0.16 | 0.09 | 0.16 | 0.01 |  |
| Monarchy | -0.37 | 0.29 | -0.41 | 0.30 | 0.36* | 0.17 | 0.36* | 0.01 |  |
| **Intercept** | 3.44*** | 0.29 | 3.52*** | 0.29 | 2.28*** | 0.21 | 2.33*** | 0.21 |  |
| **N** | 146,010 |  | 146,010 |  | 145,705 |  | 145,705 |  |  |
| **Random Effects** | 0.28 | 0.03 | 0.29 | 0.04 | 0.20 | 0.03 | 0.20 | 0.03 |  |
| **Random Effects (Age)** | 0.004 | 0.0005 | 0.004 | 0.001 | 0.003 | 0.0004 | 0.003 | 0.0004 |  |

Notes. SE = Standard error. Model applied robust standard errors. Models control for waves of the study, gender, education, urban residence, membership in a voluntary organization, employment status, political interest, GNI (logged), and system of government. * p<0.05, ** p<0.01, *** p<0.001, † < 0.10.

**Supplementary Table 2: Multilevel analyses of Age, Political context, and Political participation (all covariates) (continued)**

| **Variables** | **Protest** | | **Protest** | |
| --- | --- | --- | --- | --- |
|  | **Model 5** | | **Model 6** | |
|  | **Coef. (p)** | **Std. er.** | **Coef. (p)** | **Std. er.** |
| **Freedom Index** |  |  |  |  |
| Not free (Ref) |  |  |  |  |
| Fairly free | 0.17** | 0.07 | -0.15 | 0.14 |
| Free | -0.20** | 0.07 | -0.49** | 0.15 |
| **Age** | -0.006 | 0.004 | 0.01** | 0.004 |
| **Age-squared** | -0.00007^†^ | 0.00004 | -0.00007^†^ | 0.00004 |
| **Age X Freedom status** |  |  |  |  |
| Age X Not Free (Ref) |  |  |  |  |
| Age X Partly Free |  |  | 0.009* | 0.003 |
| Age X Free |  |  | 0.008* | 0.003 |
| **Study round** |  |  |  |  |
| Round 5 (Ref) |  |  |  |  |
| Round 6 | 0.02 | 0.03 | 0.02 | 0.03 |
| Round 7 | 0.33*** | 0.03 | 0.32*** | 0.03 |
| **Gender** |  |  |  |  |
| Male (Ref) |  |  |  |  |
| Female | -0.28*** | 0.02 | -0.28*** | 0.02 |
| **Education** |  |  |  |  |
| None (Ref) |  |  |  |  |
| Primary | 0.32*** | 0.03 | 0.32*** | 0.03 |
| Secondary | 0.43*** | 0.03 | 0.43*** | 0.03 |
| University | 0.68*** | 0.03 | 0.68*** | 0.03 |
| **Place of residence** |  |  |  |  |
| Rural residence (Ref) |  |  |  |  |
| Urban residence | 0.15*** | 0.02 | 0.15 | 0.02*** |
| **Voluntary association** |  |  |  |  |
| Not a member (Ref) |  |  |  |  |
| Inactive | 0.64*** | 0.03 | 0.64*** | 0.03 |
| Active | 0.87*** | 0.02 | 0.87*** | 0.02 |
| **Employed status** |  |  |  |  |
| Unemployed (Ref) |  |  |  |  |
| Employed | 0.02 | 0.02 | 0.02 | 0.02 |
| **Political interest** |  |  |  |  |
| Not interested (Ref) |  |  |  |  |
| Not very interested | 0.29*** | 0.03 | 0.29*** | 0.03 |
| Somewhat interested | 0.53*** | 0.03 | 0.53*** | 0.03 |
| Very interested | 0.76*** | 0.03 | 0.76*** | 0.03 |
| Log GNI | 0.03 | 0.10 | 0.05 | 0.09 |
| **System of government** |  |  |  |  |
| Presidential (Ref) |  |  |  |  |
| Semi-Parliamentary | 0.25 | 0.20 | 0.25 | 0.20 |
| Parliamentary | 0.44 | 0.24 | 0.44 | 0.24 |
| Monarchy | -0.34 | 0.46 | -0.23 | 0.46 |
| **Intercept** | -3.46*** | 0.75 | -3.36*** | 0.78 |
| **N** | 143,604 |  | 143,604 |  |
| **Random Effects** | 0.72 | 0.1 | 0.70 | 0.10 |
| **Random Effects (Age)** | 0.01 | 0.001 | 0.01 | 0.001 |

Notes. SE = Standard error. Model applied robust standard errors. Models control for waves of the study, gender, education, urban residence, membership in a voluntary organization, employment status, political interest, GNI (logged), and system of government. * p<0.05, ** p<0.01, *** p<0.001, † < 0.10.

**Supplementary Table 3: Factor loadings for indicators of political participation**

| **Indicators of** **Political participation** | Factor 1 | Factor 2 |
| --- | --- | --- |
| Attending political rallies | 0.75 |  |
| Working for political candidates | 0.75 |  |
| Political Partisanship | 0.40 |  |
| Attending community meetings |  | 0.83 |
| Raising an issue |  | 0.83 |
| Protest or demonstrations |  | 0.27 |
| **Kaiser Meyer Olkin Score – 0.68** |  |  |
